# PET/CT-based target volume definition in involved-site radiotherapy for treatment of early-stage nodal follicular lymphoma

**DOI:** 10.1007/s00066-024-02356-x

**Published:** 2025-01-14

**Authors:** Antje Wark, Ji-Young Kim, Elena Mavriopoulou, Christian la Fougère, Thomas Wiegel, Christian W. Scholz, Christian Baues, Minglun Li, Thomas Gauler, Stephanie E. Combs, Klaus Herfarth

**Affiliations:** 1 Department of Radiation Oncology, Heidelberg University Hospital, Heidelberg, Germany; 2 Heidelberg Institute of Radiation Oncology (HIRO), Heidelberg, Germany; 3 Department of Nuclear Medicine, Heidelberg University Hospital, Heidelberg, Germany; 4 Institute of Nuclear Medicine und Clinical Molecular Imaging, University Hospital Tübingen, Tübingen, Germany; 5 Department of Radiation Oncology, Ulm University Hospital, Ulm, Germany; 6 Department of Hematology and Oncology, Vivantes Klinikum Am Urban, Berlin, Germany; 7 Clinic and Polyclinic for Radiation Oncology, Cyberknife and Radiation Therapy, Universitätsklinikum of Cologne, Cologne, Germany; 8 Department of Radiation Oncology, University Hospital LMU Munich, Munich, Germany; 9 Department of Radiation Therapy, University Essen, Essen, Germany; 10 Department of Radiation Oncology, Hospital of the Technical University Munich, Munich, Germany

**Keywords:** Staging, Stage migration, Radiation therapy, GAZAI

## Abstract

**Purpose:**

Recent advancements in imaging, particularly 18F-fluorodeoxyglucose positron-emission tomography–computed tomography (FDG-PET/CT), have improved the detection of involved lymph nodes, thus influencing staging accuracy and potentially treatment outcomes. This study is a post hoc analysis of the GAZAI trial data to evaluate the impact of FDG-PET/CT versus computed tomography (CT) alone on radiation target volumes for involved-site radiotherapy (IS-RT) in early-stage follicular lymphoma (FL).

**Methods:**

All patients in the GAZAI trial underwent pretherapeutic FDG-PET/CT examinations, which were subject to central quality control. Lymph nodes with pathological metabolism were assessed for CT morphology. Differential regional involvement and the impact on radiation target volume for IS-RT were compared between PET/CT-based to solely CT-based staging.

**Results:**

In 54 patients with PET-positive lymph nodes after initial surgery, 170 involved lymph nodes were identified in total. FDG-PET/CT identified additionally involved lymph nodes not detected by CT in 61% of the patients, leading to a significant change in radiation treatment fields for 30% of the cohort. Only 58% of all involved lymph nodes exhibited pathological CT morphology. The findings were robust across different Deauville score thresholds and CT morphological metrics.

**Conclusion:**

The findings confirm the essential role of FDG-PET/CT in accurately defining the radiation volume for treatment of early-stage follicular lymphomas with radiotherapy. These results support the integration of FDG-PET/CT into the standard diagnostic pathway and its inclusion in the service catalogue of statutory health insurance, emphasizing its importance for optimal treatment planning and the potential impact on patient outcomes.

## Introduction

Follicular lymphoma is a B cell lineage disease and represents the most common form of indolent lymphoma. In the limited disease stage, excellent disease control rates can be achieved using curatively intended radiotherapy [1–4]. Determination of the disease stage through staging examinations is crucial for subsequent treatment decisions.

In recent decades, the extension of radiation volumes in the treatment of indolent lymphomas has undergone multiple changes. According to the recommended involved-site radiation therapy (IS-RT), only the involved lymph nodes with an adequate margin should be irradiated [4–7]. Compared to historical treatment concepts, IS-RT could potentially reduce the radiation dose in the surrounding healthy tissue and thus the occurrence of side effects and late effects [8, 9]. For planning of IS-RT, correct definition of the radiation fields is essential for treatment success. The International Lymphoma Radiation Oncology Group (ILROG) recommends performing an 18F-fluorodeoxyglucose (FDG) positron-emission tomography–computed tomography (PET/CT) examination and an additional contrast-enhanced CT examination for the definition of radiation volumes [5]. The radiation fields of the involved-site irradiation should encompass all lymph nodes with increased metabolism and surrounding lymph nodes with suspicious morphology. The margin should be chosen considering adjacent non-primarily suspicious lymph nodes and the anatomical boundaries of the respective lymph node region.

Regardless of the World Health Organization (WHO) grade, FDG-PET/CT shows high sensitivity and specificity in depicting follicular lymphomas [10]. Initial FDG-PET/CT in follicular lymphoma (FL) leads to an estimated upstaging in 10 to 60% of patients, especially in limited-stage disease identified by CT [11, 12]. Previous retrospective studies have reported excellent outcomes for PET-staged patients with limited-stage FL after definitive radiotherapy [2, 13]. Going further, this study aims to investigate the effect of FDG-PET/CT in comparison to CT alone for target volume definition in IS-RT.

The GAZAI trial was a multicenter, nonrandomized, noncontrolled phase II trial which examined the effectiveness of IS-RT with 2 × 2 Gy in combination with the fully humanized anti-CD20 antibody obinutuzumab in patients with early-stage (I and II) nodular FL (grades 1 and 2) [14]. FDG-PET/CT for staging was mandatory and centrally evaluated. The aim of this analysis of the prospectively acquired data from the GAZAI trial was to investigate the effects of PET/CT in comparison to CT-only morphological staging on target volume definition for involved-site radiotherapy.

## Materials and methods

A post hoc analysis was conducted in patients of the GAZAI trial with nodal follicular lymphomas of WHO grades 1 and 2 in limited stages who were treated with radioimmunotherapy. The presence of stage I or II disease according to the Ann Arbor classification was validated in all included patients by PET/CT. Patient accrual for this trial was designed to assume 15% upstaging to advanced stages III and IV after initial FDG-PET/CT, and a fraction of 30% of patients to be classified as PET-negative with no remaining PET-positive lymph nodes upon initial surgery for diagnosis [3]. All stage I and II patients with PET-positive lymph nodes after initial surgery were included for this analysis.

According to the study protocol, all patients underwent a pretherapeutic FDG-PET/CT examination, which was subjected to central quality control. The evaluation was based on the Deauville five-point scale, with the maximum standard uptake value (SUVmax) of a representative liver area being used as a comparative value, analogous to Barrington et al. [15]. Lymph nodes with a Deauville score ≥ 3 were considered to exhibit pathological metabolic activity as per study protocol in the GAZAI study.

In all lymph nodes with correspondingly increased metabolism, CT morphology was examined. Following the recommendations of the ILROG and the International Working Group [6], lymph nodes with a short-axis diameter (SAD) of more than 10 mm measured in the transverse plane were defined as pathological. We assessed both absolute and patient-specific numbers of PET-positive lymph nodes without pathological CT morphology.

Furthermore, the positions of PET-positive lymph nodes were also assessed respective to the classical lymph node regions [16, 17]. For IS-RT, the gross target volume (GTV) should include metabolically and/or morphologically suspicious manifestations of lymphoma. Moreover, distinct nodal volumes separated by ≥ 5 cm could be treated with separate fields [18]. Thus, the impact of PET-positive, CT-morphologically nonsuspicious lymph nodes on radiation target volumes was assessed by quantifying significant extensions of the target volume (hitherto termed sites). These were defined as extensions of the same target volume > 5 cm, e.g., due to multiple adjacent PET-positive lymph nodes encompassed in one GTV or extensions of the target volume by separate fields due to distinctly involved lymph nodes located > 5 cm apart. For both regional and site involvement, patient-specific comparisons were made for PET staging and pathological CT morphology only.

In order to account for different practices in previous studies [11, 13], analyses were repeated with two independent modifications. Firstly, lymph nodes were classified for pathological CT morphology by a long-axis diameter (LAD) ≥ 15 mm instead of a SAD of ≥ 10 mm. Secondly, PET-positive lymph nodes were filtered for a Deauville score ≥ 4 instead of ≥ 3. Statistical significance of differences between LAD vs. SAD and DS ≥ 4 vs. ≥ 3 on the fraction of PET-positive lymph nodes without pathological CT morphology was assessed by McNemar’s chi-squared test and one-sample proportions test, respectively.

## Results

The GAZAI study yielded 54 patients with follicular lymphoma with residual PET-positive lymph nodes after initial surgery. A total of 170 lymph nodes with increased FDG uptake (Deauville score ≥ 3) were analyzed. The median number of PET-positive lymph nodes per patient was 2 (IQR: 1–4, range: 1–18). Twenty-four patients (44%) presented with supradiaphragmatic and 30 patients with infradiaphragmatic disease.

Of all lymph nodes with increased metabolism, 98 (58%; Table 1) simultaneously exhibited pathological CT morphology, defined as a short-axis diameter ≥ 10 mm. Thus, 42% of the involved lymph nodes remained undetected in CT staging.

**Table 1:** Positron-emission tomography (PET)-positive lymph nodes of patients in the GAZAI study

	*n*	%
Lymph nodes with pathological FDG uptake	170	100
Fraction of PET-positive lymph nodes with pathological CT morphology	98	58

*FDG* ^18^F-Flourdesoxyglucose, *CT* Computed tomography

FDG-PET/CT enabled identification of CT morphologically nonsuspicious lymph nodes with suspicious FDG uptake in a total of 33 of the 54 analyzed patients (61%; Table 2). In 16 patients, one additional lymph node with increased FDG uptake was identified. Two, three, and four additional lymph nodes were identified in 8, 6, and 1 patient(s), respectively. In 2 patients, up to 7 additional lymph nodes were identified.

**Table 2:** Additional regional and site involvement in patients detected with positron-emission tomography (PET)-positive lymph nodes

	*n*	%
Number of patients with PET-positive lymph nodes	54	100
Fraction of patients with ≥ 1 additionally involved lymph node	33	61
Fraction of patients with ≥ 1 additionally involved region	18	33
Fraction of patients with a significantly modified RT target volume	16	30

In order to assess the impact of PET-positive lymph nodes without pathological CT morphology on the radiation target volume, we assessed their impact on regional involvement as well as on additional sites to which the IS-RT radiation target volume was extended. In 18 patients (33%), at least one additional lymphatic region was involved, of whom 15 had one and three had two additional lymph node regions involved. The numbers of involved regions staged by CT morphology and FDG uptake are listed in Table 3. Radiation target volume had to be expanded in 16 patients (30%) in order to cover additional involved sites detected by FDG-PET/CT. Due to proximity to the other involved nodes for 2 patients, the additionally detected involved regions could be adequately covered in one involved-site target volume. Thus, these were not regarded as patients with a modified radiation target volume.

**Table 3:** Number of involved lymphatic regions staged by CT versus FDG/PET-CT

Number of involved regions (*N*)	Number of patients (*n* = 54)			
	CT		FDG-PET/CT	
	*n*	%	*n*	%
1	33	61	23	43
2	17	32	20	37
3	4	7	7	13
4	0	0	4	7

**Fig. 1:**
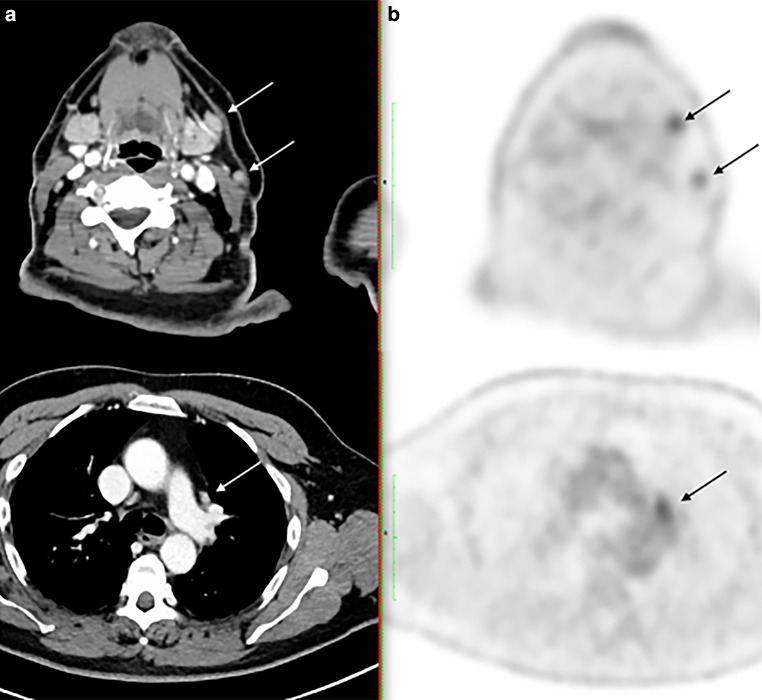
Assessment of CT morphology (**a**) and FDG uptake in PET (**b**) of lymph nodes in a patient with stage II follicular lymphoma. This particular patient had a lymph node resected to establish the diagnosis in the right cervical region. FDG-PET/CT led to extension of the radiation fields to include the left cervical (top; SUVmax: 7.0 and 4.4) and mediastinal region (bottom; SUVmax: 6.1). The arrows mark PET-positive lymph nodes. *CT* computed tomography; *PET* positron emission tomography; *FDG* ^18^F-Flourdesoxyglucose; *SUVmax* maximal standard uptake value

As an example, the evaluation of metabolism and CT morphology of lymph nodes for a patient with stage II follicular lymphoma is depicted in Fig. 1.

We repeated the analysis by defining pathological CT morphology of lymph nodes by a long-axis diameter of ≥ 15 mm [11]. By this definition, 91 out of 170 PET-positive lymph nodes (54%) did not exhibit simultaneous pathological CT morphology. With this metric, additional regional and site involvement occurred in 21 and 18 patients, respectively. The difference in the fraction of PET-positive lymph nodes without pathological CT morphology was not statistically significant compared to defining pathological CT morphology as a short-axis diameter ≥ 10 mm (*p* = 0.296; McNemar’s test).

In addition, the analysis was repeated by analyzing lymph nodes with a Deauville score ≥ 4. In this case, six of the 54 analyzed patients would have been considered PET negative. In total, 135 PET-positive lymph nodes were PET-positive by this definition. As before, raising the DS threshold did not lead to meaningful changes in assessed ratios: 85 of 135 (63%) lymph nodes with increased FDG uptake showed pathological CT morphology (*p* = 0.370; one-sample proportions test). Assessing the impact of PET-positive (with a Deauville score ≥ 4) lymph nodes without pathological CT morphology on radiation target volumes, in 20 of 48 patients (37%), at least one additional region was involved, and in 23 patients (43%), additional sites led to extension of RT fields compared to a solely CT-based analysis.

## Discussion

Recently, a comprehensive review of the clinical practice of using PET/CT imaging in radiation treatment planning was published by a German expert group, emphasizing the value of PET/CT as a tool for radiation therapy planning of various tumor entities in different clinical scenarios. The necessity of adjustment of reimbursement regulations by German statutory health insurance providers for corresponding examinations for internationally accepted indications was discussed in this context [19].

The high sensitivity and specificity of FDG-PET/CT examinations in the initial staging and restaging of non-Hodgkin lymphoma have been demonstrated in various publications [10, 20]. An analysis of the cohort from the Italian multicentric FOLL05 study showed that FDG-PET/CT identified additional involved lymph nodes in 32% of patients with follicular lymphoma (stage II to IV), with the greatest effect observed in patients in the limited stage, resulting in upstaging in 62% of these patients [11]. Our study did not investigate upstaging, as we only analyzed patients who were classified as limited stage after centralized PET review. Nonetheless, we identified additionally involved lymph nodes in 61% of patients, which is a higher proportion than in the FOLL05 study. This is likely a result of our cohort containing stage I and II patients only and thus a corollary of the observation of a higher effect on upstaging in early stages in the FOLL05 study due to FDG-PET/CT.

Since the FDG-PET/CT examinations within the prospective GAZAI study underwent central evaluation and the CT data were assessed based on standardized criteria, the study is based on homogeneous data despite its multicentric cohort.

This analysis confirms the superiority of FDG-PET/CT for detection of involved lymph nodes. Conventional CT staging could only detect all involved lymph nodes in around 40% of patients in this study. This work furthermore demonstrates the implications for radiotherapy. In a third of patients, PET/CT staging led to significant modification of the radiation target volume for IS-RT. These conclusions were robust across different definitions of pathological CT morphology of lymph nodes and threshold levels of metabolic activity.

As previously stated, several studies have shown that most relapses after radiotherapy in patients with limited-stage follicular lymphoma occur at distant sites with low rates of local or marginal recurrence [2, 4, 7, 21, 22]. These findings provided the rationale for prospective studies which evaluate low-dose radiotherapy in combination with systemic therapy [3, 14]. Nonetheless, reduced radiation target volumes are associated with higher anatomic uncertainty, which may increase the local relapse risk [23, 24]. PET-based delineation of the radiation target volume may help to decrease this variability [25] and would seem to be crucial.

The results from this study indicate that FDG-PET/CT in patients with follicular lymphoma not only significantly impacts staging but also influences the extent of radiation fields in involved-site radiation treatment of follicular lymphomas. Thus, inclusion of PET/CT examination is fundamental for the adequate treatment of early-stage follicular lymphomas.

## Conclusion

The present data confirm that in the curative treatment of follicular lymphomas in the limited stage with radiotherapy, an FDG-PET/CT examination is essential for correct definition of the radiation volume. The results of this study support the demand for inclusion of the corresponding FDG-PET/CT examination for this indication in the catalogue of the German health insurance providers.
